# Male partner testing and sexual behaviour following provision of multiple HIV self‐tests to Kenyan women at higher risk of HIV infection in a cluster randomized trial

**DOI:** 10.1002/jia2.25515

**Published:** 2020-06-26

**Authors:** Sue Napierala, Elizabeth F Bair, Noora Marcus, Perez Ochwal, Suzanne Maman, Kawango Agot, Harsha Thirumurthy

**Affiliations:** ^1^ Women’s Global Health Imperative RTI International San Francisco CA USA; ^2^ Department of Medical Ethics and Health Policy Perelman School of Medicine University of Pennsylvania Philadelphia PA USA; ^3^ Impact Research and Development Organization Kisumu Kenya; ^4^ Department of Health Behavior Gillings School of Global Public Health University of North Carolina at Chapel Hill Chapel Hill NC USA

**Keywords:** HIV self‐testing, women at higher risk, male partner testing, couples testing, secondary distribution

## Abstract

**Introduction:**

Without significant increases in uptake of HIV testing among men, it will be difficult to reduce HIV incidence to disease elimination levels. Secondary distribution of HIV self‐tests by women to their male partners is a promising approach for increasing male testing that is being implemented in several countries. Here, we examine male partner and couples testing outcomes and sexual decision making associated with this approach in a cluster randomized trial.

**Methods:**

We examined data from women at higher risk of HIV participating in the intervention arm of an ongoing pair‐matched cluster randomized trial in Kenya. HIV‐negative women ≥18 years who self‐reported ≥2 partners in the past month were eligible. Participants received self‐tests at enrolment and three‐monthly intervals. They were encouraged to offer tests to sexual partners with whom they anticipated condomless sex. At six months, we collected data on self‐test distribution, male partner and couples testing, and testing and sexual behaviour in the three most recent transactional sex encounters. We used descriptive analyses and generalized estimating equation models to understand how sexual behaviour was influenced by self‐test distribution.

**Results:**

From January 2018 to April 2019, 921/1057 (87%) participants completed six‐month follow‐up. Average age was 28 years, 65% were married, and 72% reported income through sex work. Participants received 7283 self‐tests over six months, a median of eight per participant. Participants offered a median three self‐tests to sexual partners. Of participants with a primary partner, 94% offered them a self‐test. Of these, 97% accepted the test. When accepted, couples testing was reported among 91% of participants. Among 1954 transactional sex encounters, 64% included an offer to self‐test. When offered self‐tests were accepted by 93% of partners, and 84% who accepted conducted couples testing. Compared to partners with an HIV‐negative result, condom use was higher when men had a reactive result (56.3% vs. 89.7%, *p* < 0.01), were not offered a self‐test (56.3% vs. 62.0%, *p* = 0.02), or refused to self‐test (56.3% vs. 78.3, *p* < 0.01).

**Conclusions:**

Providing women with multiple self‐tests facilitated male partner and couples testing, and led to safer sexual behaviour. These findings suggest secondary distribution is a promising approach for reaching men and has HIV prevention potential.

**Clinical Trial Number:** NCT03135067.

## 1 INTRODUCTION

In much of eastern and southern Africa, HIV incidence remains high despite the scale‐up of promising biomedical and behavioural prevention interventions [1]. The UNAIDS 95‐95‐95 fast track goals to end the AIDS epidemic by 2030 provide tangible targets for testing and treatment that can substantially reduce HIV incidence in the region [2]. However, these goals may be compromised by disparities in engagement in HIV services among certain subpopulations. Men in sub‐Saharan Africa in particular continue to be less engaged in services [3, 4, 5]. Despite impressive increases in knowledge of HIV status across the region, uptake of HIV testing remains low among men. Of the 21 countries in eastern and southern Africa region, as designated by UNAIDS, all but one report more women than men testing in the past 12 months [6]. This differential engagement in HIV services represents a public health inequity, and contributes to high risks of HIV infection among adolescent girls and young women. Without significant increases in uptake of HIV testing among men, it will be difficult to achieve the 95‐95‐95 goals and reduce HIV incidence to disease elimination levels [1].

The privacy, convenience and autonomy that oral fluid‐based HIV self‐testing provides has the potential to overcome many of the barriers to HIV testing, including those cited by men. This strategy has high acceptability among men; and in community‐based distribution of self‐testing, uptake by men has often been roughly equal to that of women [7, 8, 9]. The World Health Organization has recommended the scale‐up of HIV self‐testing as an alternative testing strategy, and a number of countries have begun to make self‐tests available [10]. Models for delivering self‐tests to men, and others less likely to use standard HIV testing services (HTS), are being considered and a promising strategy is the provision of multiple self‐tests to women at higher risk so they can voluntarily initiate partner or couples testing [11]. This strategy of “secondary distribution” has the potential to generate multiple HIV prevention benefits, including promotion of male partner testing, results disclosure and facilitation of safer sexual behaviours. This has been included as a potential distribution strategy in Kenya’s 2017 operational manual for HIV self‐testing [12]. Research in Kenya and elsewhere has demonstrated that secondary distribution is acceptable and feasible, and that women from different settings, including antenatal clinics and sex workers, are able to distribute self‐test kits to their sexual partners [11, 13, 14]. While this approach is being implemented in several countries, data from large‐scale studies on partner and couples testing outcomes are lacking, and there are few data on changes in sexual decision making following secondary distribution [11, 13, 15].

To assess the HIV prevention potential of this approach, we are conducting a cluster randomized trial (cRCT) of secondary distribution of self‐tests by women at higher risk in Kenya (NCT03135067). The study is being conducted in beach, peri‐urban and urban communities in Siaya County, Kenya [16, 17]. HIV prevalence in Siaya is among the highest in Kenya, at 21% [18]. Despite progress in reducing the spread of HIV in the region overall, HIV incidence remains persistently high in these communities [19, 20, 21]. The prominence of multiple partnerships and transactional sex in this region has been widely documented [21, 22, 23, 24]. Here we describe outcomes for women in the intervention arm only of the pair‐matched cRCT following six months of self‐test provision. We explored self‐test kit distribution patterns and male partner testing uptake, results disclosure, couples testing, as well as testing and sexual behaviour data from the three most recent transactional sex encounters.

## 2 METHODS

### 2.1 Study design and participants

The study is being conducted in a total of 66 geographic clusters in Siaya County, Kenya. Each cluster consists of one or more nearby beach communities along Lake Victoria where fishing drives the local economy as well as market centres containing hotspots (bars and hotels) where transactional sex is common. Clusters were defined after a comprehensive mapping of beach communities and hotspots in the study region. Nearby beach communities were consolidated into a single cluster, as were hotspots that were located near each other. Clusters were matched on the basis of spatial proximity, population size and type (hotspot or beach community) and pairs of clusters were randomized in a 1:1 ratio to an intervention arm in which participants received multiple self‐tests or to a comparison arm in which participants were given referral cards for clinic‐based HIV testing and counselling to distribute to their male sexual partners. Computer‐generated randomization was used to determine study arm assignment of clusters. After randomization, the study team conducted household surveys to prepare a list of adult women in each cluster who were potentially eligible. Women were then selected from the list at random for recruitment into the study. Based on power calculations for the primary cRCT outcome of HIV incidence, we sought to enrol about 30 participants in each cluster.

Upon recruitment from each cluster women were screened for eligibility. Eligibility criteria were as follows: age ≥18 years, residing in the study area and intending to stay there for at least 24 months, ownership of a mobile phone, HIV‐negative and self‐reporting ≥2 male partners in the past four weeks. Eligible participants were enrolled after providing written informed consent in their preferred language (English, Swahili or Dholuo). Prior to enrolment, participants underwent standard HIV testing according to national algorithms to determine HIV status [25]. Participants completed an interviewer‐administered questionnaire at baseline that collected information on a range of topics including demographics, sexual behaviour and HIV testing history. Screening and follow‐up visits were conducted at study sites established within the study communities, and data collection instruments were administered in the preferred language of the participant.

Participants in the intervention arm were trained on the use of oral fluid‐based rapid HIV self‐tests (OraQuick Rapid HIV‐1/2 antibody tests; OraSure Technologies, Bethlehem, PA, USA). They were counselled on how to discuss HIV self‐testing with male sexual partners and on the importance of using their own discretion and assessing the risk of intimate partner violence (IPV) when deciding whether to offer a self‐test to a sexual partner. Participants were encouraged to offer tests to their male primary partner and to any other male sexual partners with whom unprotected sex was likely. They received five self‐tests at enrolment. Self‐test kits included written and pictorial instructions for use, including information on results interpretation and a list of clinics in the area where they could confirm their test result and seek post‐test services. Research assistants contacted participants at three‐monthly intervals and provided them additional self‐tests, as needed. Participants were also able to receive self‐tests in‐between three‐monthly intervals by contacting research assistants. At six months, we collected follow‐up data via interviewer‐administered questionnaire on self‐test distribution, partner uptake, results disclosure and couples testing – defined as testing by the participant and her partner testing together at the same time – and sexual behaviour. We also obtained testing and sexual behaviour information on participants’ three most recent transactional sex encounters. Transactional sex was defined as sex in exchange for money, goods or services, in line with the Joint United Nations Programme on HIV/AIDS definition [26]. The study is ongoing, with participants completing follow‐up assessments and HIV testing every six months for a duration of up to 24 months. All questionnaires and study materials were in English, Kiswahili or Dholuo, based on participant preference.

### 2.2 Outcomes and measures

Key outcomes assessed in this analysis were the self‐reported number of self‐tests distributed by participants and the proportion distributed to a male sexual partner. We evaluated, through participant self‐report, the proportion of male partners who accepted self‐tests when offered, and who disclosed their results to the participant. Test results were categorized as HIV‐positive, HIV‐negative, indeterminate or unknown. We also evaluated couples testing, as indicated by participant report that they tested together with a sexual partner. Sexual decision making among women who reported transactional sex was another key outcome. Using the data obtained on the three most recent transactional sex encounters, we examined the association between self‐testing outcomes and whether or not participants had sex with the transaction sex partner as well as whether a condom was used in the encounter. We explored these outcomes by four categories of transactional sex partners: clients who had a reactive self‐test, clients who tested HIV‐negative, clients who refused the self‐test and clients who were not offered a self‐test.

### 2.3 Statistical analyses

We conducted descriptive analyses for all outcomes among intervention arm participants at the six‐month visit. We focused on self‐test distribution to sexual partners, partner testing and result, and sexual behaviour change. To understand how sexual behaviour was influenced by distribution of self‐tests to sexual partners we used unadjusted generalized estimating equation models. We clustered encounters by participant to compare participant reporting of condom use with sexual partners by use of HIV self‐tests, HIV‐negative result, or reactive result. All data were analysed using Stata 15.1 (StataCorp, College Station, TX, USA).

### 2.4 Ethical considerations

The study received ethics approval from the institutional review boards at the University of Pennsylvania and the University of North Carolina at Chapel Hill as well as the Maseno University Ethics Review Committee. Eligible women who wished to participate provided written informed consent in their preferred language (English, Swahili or Dholuo) prior to initiation of any study procedures.

## 3 RESULTS

A total of 1057 participants from 33 clusters were enrolled in the intervention arm of the study between June 2017 and August 2018. A total of 265 women were enrolled in eight beach community clusters and 792 from 25 hotspots. Between January 2018 and April 2019, 921 (87%) participants completed the six‐month follow‐up visit and were included in this analysis. Participants’ average age was 28 years, with the majority (65%) being married and 63% having at least a primary school education (Table 1). Sex work was the primary source of income for 14% of participants, and an additional 58% reported earning some income through sex work.

**Table 1:** Baseline characteristics of participants randomized to the intervention arm who completed the six‐month follow‐up (N = 919^a^)

Variable	N (%)
Demographics	
Age, mean (SD)	27.6 (6.9)
Education	
Some primary or less	303 (33.0)
Primary	276 (30.0)
Some secondary	168 (18.3)
Secondary/high school or more	172 (18.7)
Marital status	
Married and/or cohabitating	597 (65.0)
In a relationship, but not married or living together	91 (9.9)
Single	155 (16.8)
Divorced or widowed	76 (8.3)
Primary source of income	
Sales and service	309 (33.6)
Sex work	129 (14.0)
Unskilled manual	128 (13.9)
Fishing/fish trade	98 (10.7)
Agriculture	50 (5.4)
Unemployed	53 (5.8)
Other	151 (16.4)
Refused	1 (0.1)
Sex work is another source of income	531 (57.8)
Typical one‐month income in U.S. $, median (IQR)	30 (20, 60)
Household size, median (IQR)	5 (4, 6)
Male sexual partners and sexual behaviour	
Number of sexual partners in the past month, median (IQR)	2 (2, 3)
Used condom during last sexual encounter	335 (36.5)
Ever engaged in transactional sex^b^	869 (94.6)
Number of transactional sex partners in the past month, median (IQR)^c^	2 (1, 2)
Used condom with most recent transactional sex partner during vaginal or anal sex^d^	440 (51.3)
Experienced any type of intimate partner violence in the past 12 months	475 (51.6)

^a^ 921 women in the intervention arm completed the six‐month follow‐up questionnaire, however baseline questionnaire data for two of those individuals was lost

^b^ Transactional sex defined as sex in exchange for money, goods, food, housing, or services

^c^ Among 868 participants reporting transactional sex in the past month

^d^ Among 858 encounters involving vaginal or anal sex. IQR, inter‐quartile range

### 3.1 HIV self‐test distribution

**Figure 1:**
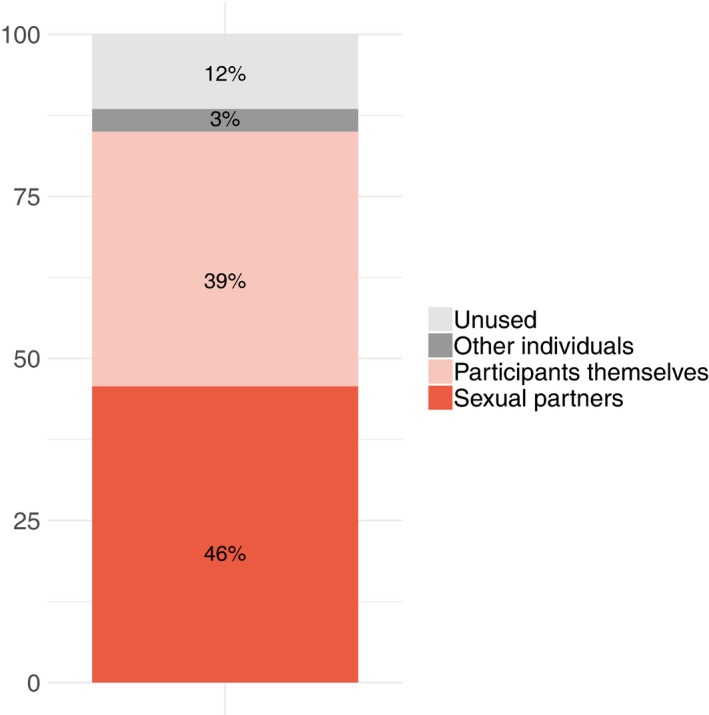
Use of 292 HIV self‐test kits distributed to 922 participants.

Over the six months of follow‐up, participants received a total of 7283 self‐test kits for distribution, a mean (IQR) of eight self‐tests per person (7,9). Figure 1 shows self‐test kit distribution by participants. All sexual partners in this study, including primary and transactional sex partners, were male. Participants distributed a total of 3327 (46%) of self‐tests to sexual partners, a mean (IQR) of 3 (2,5) per person. Participants reported that 153 partners disclosed reactive results, equalling 4.6% (153/3327) of all tests distributed which could be confirmed as reactive, and an average of 0.17 HIV‐positive partners being identified per participant. There were 253 (3%) self‐tests distributed to other individuals, that is non‐sexual partners, and 2862 (39%) self‐tests were used by participants themselves. The remaining 841 (12%) self‐tests were unused.

### 3.2 Self‐testing with primary partners

**Figure 2:**
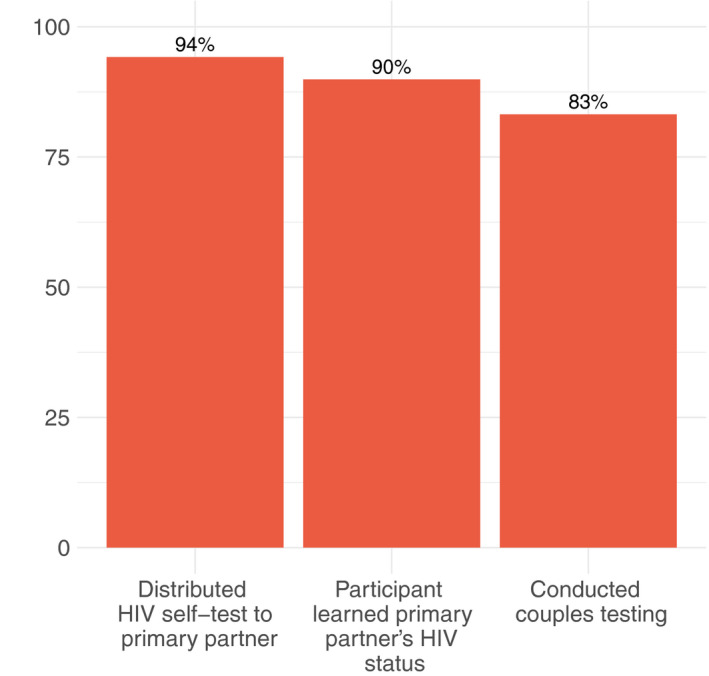
Primary partner and couples testing (N = 891).

Of 890 participants with a primary partner, 838 (94%) offered their primary partner a self‐test (Table 2). A total of 813 (97%) accepted the self‐test. Among primary partners who accepted a self‐test, 800 (98%) shared their test results with the participant, with 15 (2%) reporting a reactive result. Couples testing was reported by 740 (91%) participants whose primary partner accepted a self‐test, and in all 83% of all participants with a primary partner conducted couples testing with them (Figure 2).

**Table 2:** HIV self‐test use reported by participants among their male primary partners (N = 890)

Variable	N (%)
Age difference, primary partner age – participant age, median (IQR)^a^	5 (2, 7)
Offered HIV self‐test to primary partner	838 (94.2)
Primary partner accepted the self‐test^b^	813 (96.9)
Participant didn’t know if primary partner used self‐test	7 (0.9)
Primary partner used self‐test, but participant didn’t learn result	6 (0.7)
Primary partner test was reactive	15 (1.8)
Primary partner test was HIV‐negative	785 (96.6)
Participant conducted couples testing with primary partner^c^	740 (91.0)

^a^ N = 851. 39 participants did not report primary partner age

^b^ Among 839 participants who offered a HIV self‐test to their primary partner

^c^ Among 813 participants whose partner accepted the self‐test. Couples testing is defined as participant‐reported testing together with a sexual partner.

### 3.3 Self‐testing with transactional sex partners

A total of 870 (94%) participants reported having transactional sex during the study period, and we asked these participants about their three most recent transactional sex encounters. Participants reported on a total of 1954 transactional sex encounters involving vaginal and/or anal sex in the past six months (Table 3). Of those, 1256 (64%) encounters included an offer of a self‐test to the partner and 1173 (93%) partners accepted the self‐test. Among all encounters in which a self‐test was accepted, the partner disclosed their test result in 1133 (97%) encounters, and 29 (3%) results were reactive. In 987 (84%) encounters the participant and their partner tested together.

**Table 3:** Use of HIV self‐tests during transactional sex encounters with male partners involving vaginal and/or anal sex (N = 1954)

Variable	N (%)
Offered HIV‐self test to transactional sex partner	1256 (64.3)
Among those offered HIV self‐test, transactional sex partner accepted the self‐test^a^	1173 (93.4)
Participant did not learn result	21 (1.8)
Partner test was reactive	29 (2.5)
Partner test was HIV‐negative	1101 (93.9)
Partner test was indeterminate	3 (0.3)
Among those offered HIV self‐test, participant conducted couples testing with transactional sex partner^b^	987 (84.1)

^a^ Among 1256 participants who offered a self‐test to their partner. HIV test results were missing for 19 (1.5%) partners

^b^ Among 1173 transactional sex encounters where a self‐test was offered.

### 3.4 Sexual behaviour with transactional sex partners

**Figure 3:**
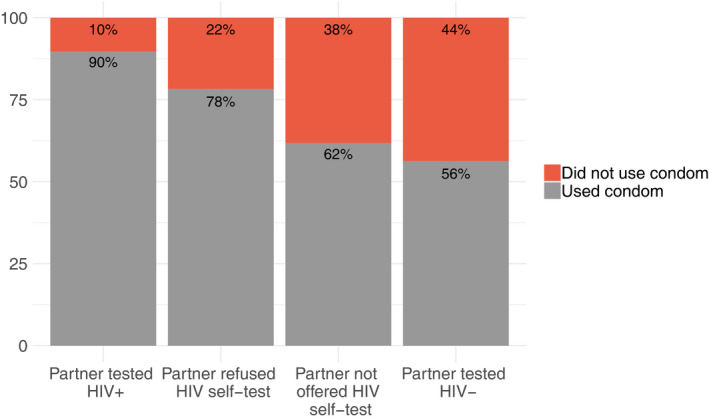
Condom use during recent transactional sex encounters (N = 1957).

Figure 3 shows condom use based on transactional sex partner uptake, utilization and self‐test result. Condom use was significantly higher with transactional sex partners who obtained a reactive versus HIV‐negative result (89.7% vs. 56.3%, *p* < 0.01). Condom use was also significantly higher with transactional sex partners who were not offered a self‐test as compared to those with a HIV‐negative result (62.0% vs. 56.3%, *p* = 0.02), or who refused to self‐test versus those with a HIV‐negative result (78.3 vs. 56.3%, *p* < 0.01).

### 3.5 Sexual behaviour with all male partners

In terms of overall sexual behaviour among study participants at six months, participants reported that the mean number of sexual partners in the past month was 2.8 at baseline and 2.2 at the six‐month follow‐up visit (Table 4). The proportion of participants who reported using a condom during their last sexual encounter was 37% at baseline and 44% at the six‐month follow‐up. A total of 131 (14%) participants reported refusing to have sex with 158 potential sexual partners because they either refused to accept a self‐test or had a reactive test result. Additionally, 107 (12%) women reported that they decided to use a condom with 141 sexual partners because they either refused the self‐test or had a reactive result.

**Table 4:** Overall sexual behaviour among study participants (N = 921)

Variable	N (%)
Number of sexual partners in the past month, median (IQR)	2 (1, 3)
Used condom during last sexual encounter	403 (43.8)
In past six months, participant declined to have sex with one or more partners because they refused to accept a HIV self‐test, or had a reactive self‐test	131 (14.2)
Total number of partners	158
In past six months, participant used a condom with one or more partners because they refused to accept a HIV self‐test, or had a reactive self‐test	107 (11.6)
Total number of partners	141

## 4 DISCUSSION

This is the first large‐scale study to evaluate self‐reported self‐test distribution patterns and partner testing outcomes in the context of secondary distribution by women at higher risk of HIV infection. The study also examines changes in sexual decision making with transactional sex partners enabled by self‐testing. Among women receiving multiple self‐tests in Kenya, nearly 50% of self‐tests provided were offered to male sexual partners and high rates of partner and couples testing were observed. This confirms findings from other research demonstrating that women can readily and capably distribute self‐tests to both their regular and transactional sex partners, and that men are willing to accept and use tests distributed by their sexual partners [11, 13, 27]. This finding is of particular relevance for women at higher risk in the study setting, as they are likely to be in contact with and distribute test kits to men who are also at high risk of HIV transmission. We found that when a test kit was offered, uptake by men was high and results disclosure between both regular and transactional sex partners was common. Furthermore, couples testing was very common, with a large proportion of the test kits provided being used by women to test together with their partner.

One of the most compelling findings in this study relates to sexual decision making with self‐tests. We found significantly higher condom utilization with transactional sex partners when a self‐test was refused and when the result was reactive, as compared with transactional sex partners who had a negative result. At the same time, we found moderately higher condom use when a self‐test was not offered as compared to a negative result as well (62% vs. 56%). While the latter finding implies less condom use with known HIV‐negative partners and therefore a potential shift in risk perception, women were encouraged to offer a self‐test to partners when they did not intend to use a condom. Furthermore, we have no information about the relative risk profiles of those men who were and were not offered self‐tests. Therefore, these data should be interpreted with caution. Among participants overall, 14% reported refusing sex, and 12% reported using a condom, with one or more partners if they were not offered or refused a self‐test. These sexual decision‐making data suggest there is substantial potential for this strategy to have HIV prevention benefits for both women and their male sexual partners.

The data available to us did not permit an accurate estimate of male HIV‐positive case identification per test kit distributed – an important consideration for programme implementation. While we know the overall number of test kits distributed to male partners, we cannot discern whether a single partner used multiple test kits. Likewise, not all men who self‐tested disclosed their test result to the participant, and there may be biases in male disclosure of a positive result to participants. Of the 3327 test kits distributed to sexual partners, a total of 153 men disclosed a reactive result to a participant. It is important to note, however, that not all partners disclosed their result and some sexual partners may have used more than one self‐test during the six‐month follow up period. We can assume that at a minimum, 4.6% of all tests distributed were confirmed reactive and that 0.17 HIV‐positive partners were identified per participant. As we cannot accurately estimate overall case identification (i.e. yield), it remains unclear how case identification among men in this intervention compares to other HTS strategies, another useful data point for self‐test programming. Subsequent analyses will compare the case identification in the intervention arm to the comparison arm in which only HTS referral cards were provided. Due to the nature of our study design and inherent confidentiality of self‐testing, we do not have an accurate sense of the demographic and behavioural characteristics of the men we are reaching. As men are targeted based on their contact with women at higher risk, it is likely that the men in this research are at high risk of HIV transmission as well. Therefore, secondary distribution by women at higher risk may be a compelling strategy to engage men in HIV testing, and potentially other HIV services.

A primary limitation of this research is the reliance on self‐reported self‐test distribution and sexual behaviour data, which has the potential for bias. A study of secondary distribution among antenatal care clients and their male partners in Kenya compared male and female reporting of couples testing and found strong agreement between partners, and therefore minimal reporting bias [28]. To explore this potential bias in the current study subsequent analyses will compare these self‐reported outcomes on partner testing and sexual behaviour to those from the comparison group that did not receive self‐tests. We will also examine the effect of the intervention on HIV incidence, which is being assessed over an average 18‐month period. Another limitation is that we did not collect data around men’s use of post‐test services, another important consideration for future programming. While women were instructed to consider offering their partner a self‐test when they anticipated having condomless sex, we cannot determine overall how many sexual encounters which were “at risk” of HIV transmission included the offer of a self‐test. However, we did ask detailed questions about self‐test use in the past three transactional sex encounters and determined that 64% of these encounters included the offer of a self‐test.

Data on the cost‐effectiveness of secondary distribution by women at higher risk as an additional HTS strategy, both for the outcome of HIV prevention among women and case identification among men, will be important to explore. These data will be collected and analysed in the context of the current study and will provide an important contribution to future research and programming. Modelling studies from Zimbabwe suggest that distribution of self‐tests to women engaged in transactional sex is an efficient strategy to avert new HIV infections and is potentially cost‐effective [29]. Additional modelling suggests that secondary distribution of self‐tests by female sex workers in Zimbabwe to their male partners also has the potential to be highly cost‐effective at identifying HIV‐positive men [30].

Other important next steps for research would be to explore from men directly their views and testing outcomes related to secondary distribution of self‐tests by female partners, particularly high‐risk female partners and in the context of transactional sex. Currently all our data on acceptability and uptake among men is derived from the female partner. The in‐depth interviews we are presently conducting among male partners of study participants will provide a necessary and complementary perspective to the findings presented here. Exploring social harms associated with secondary distribution, such as IPV, is another important area of research. This is particularly relevant given the high baseline level of IPV, with 52% of participants experiencing IPV in the previous 12 months. Programming for men, particularly in the context of secondary distribution, needs to incorporate a holistic approach to men’s health. This includes addressing unhealthy masculine ideals and IPV. We are collecting and monitoring IPV data on an ongoing basis and will be presenting comparisons of IPV in the two study arms at study completion. Likewise, assessing linkage to post‐test prevention and care by men in this context would be helpful in considering how best to implement and support secondary distribution strategies. The nature of self‐testing, and secondary distribution of self‐test kits among women at higher risk in particular, make acquiring these type of data challenging. Most strategies employed in other studies, such as encouraging reporting of self‐testing upon attending post‐test services, telephone follow‐up, or centralized database tracking, have proved imperfect measures of linkage [31]. In a study of secondary distribution among antenatal care clients in Kenya 28% of men reported going for confirmatory testing after their self‐test [28]. There is no gold standard for how to support linkage, and optimal strategies will likely be context specific, but a range of approaches have been employed such as counselling, phone reminders, hotlines, incentives, referrals slips, vouchers and home initiation of HIV care [10, 31, 32]. For men, strategies that accommodate their work or lifestyle may be particularly appropriate.

Based on the willingness of participants in this study to distribute self‐tests to their primary and transactional sex partners, the high uptake of self‐testing by male partners, high levels of results disclosure, frequent couples testing, as well as evidence of safer sexual behaviour in this context, we anticipate that secondary distribution of self‐tests by women at higher risk to male sexual partners represents a promising strategy for increasing knowledge of HIV status among men, and potentially for reduction of HIV transmission. Secondary distribution by women at higher risk could be an important addition to regional and country wide HTS strategies. Operationalizing secondary distribution of self‐tests could be an important way to ensure that men have higher access to HIV testing, and could increase couples testing and promotion of risk‐reducing behaviours. Future analyses from this ongoing study will use the randomized trial design to determine the effect of the secondary distribution intervention on male partner testing outcomes on HIV incidence among women.

## 5 CONCLUSIONS

Data obtained over a period of six months indicate that women at higher risk are willing and capable of distributing HIV self‐tests to regular and transactional male partners. Providing women at higher risk with multiple self‐tests facilitated male partner testing, and couples testing. We also demonstrated positive sexual behaviour change, based on whether a self‐test was accepted by the male partner, and what the result was. Further research is required to evaluate male partner access to post self‐test services. This includes confirmatory testing for all reactive results, and linkage across the cascade of prevention and treatment services. Also important will be consideration of what type of programming can best support men’s linkage to these services. These findings suggest that secondary distribution of self‐tests is a feasible way to reach men at high risk, who may not be aware of their HIV status, and also has considerable HIV prevention potential.

## COMPETING INTERESTS

The authors have no competing interests to declare.

## AUTHORS’ CONTRIBUTIONS

SN, HT, KA and SM contributed to study design. EB, SN and HT were involved in analysis. SN, HT, KA, SM, EB, NM and PO were involved in interpretation of results. SN, HT, KA, SM, EB, NM and PO were involved in manuscript writing.

## References

[1] UNAIDS . Global AIDS update: communities at the center. New York: Joint United Nations Programme on HIV/AIDS (UNAIDS); 2019.

[2] UNAIDS . Understanding fast‐track: accelerating action to end the AIDS epidemic by 2030. Geneva: Joint United Nations Programme on HIV/AIDS (UNAIDS); 2015.

[3] Mills EJ , Ford N , Mugyenyi P . Expanding HIV care in Africa: making men matter. Lancet. 2009;374(9686):275–6. 19632481 10.1016/S0140-6736(09)61348-9

[4] Dovel K , Yeatman S , Watkins S , Poulin M . Men's heightened risk of AIDS‐related death: the legacy of gendered HIV testing and treatment strategies. AIDS. 2015;29(10):1123–5. 26035315 10.1097/QAD.0000000000000655 PMC4454403

[5] Cornell M , McIntyre J , Myer L . Men and antiretroviral therapy in Africa: our blind spot. Trop Med Int Health. 2011;16(7):828–9. 21418449 10.1111/j.1365-3156.2011.02767.x PMC3749374

[6] Staveteig S , Wang S , Head S , Bradley S , Nybro E . Demographic patterns of HIV testing uptake in sub‐Saharan Africa. DHS comparative reports no. 30. Calverton (MD): USAID; 2013.

[7] Choko AT , Desmond N , Webb EL , Chavula K , Napierala‐Mavedzenge S , Gaydos CA , et al. The uptake and accuracy of oral kits for HIV self‐testing in high HIV prevalence setting: a cross‐sectional feasibility study in Blantyre, Malawi. PLoS Medicine. 2011;8:e1001102. 21990966 10.1371/journal.pmed.1001102 PMC3186813

[8] Johnson C , Baggaley R , Forsythe S , van Rooyen H , Ford N , Napierala Mavedzenge S , et al. Realizing the potential for HIV self‐testing. AIDS Behav. 2014;18 Suppl 4:S391–5. 24986599 10.1007/s10461-014-0832-x

[9] Hatzold K , Gudukeya S , Mutseta MN , Chilongosi R , Nalubamba M , Nkhoma C , et al. HIV self‐testing: breaking the barriers to uptake of testing among men and adolescents in sub‐Saharan Africa, experiences from STAR demonstration projects in Malawi, Zambia and Zimbabwe. J Int AIDS Soc. 2019;Suppl 1:e25244. 30907505 10.1002/jia2.25244 PMC6432104

[10] WHO . Guidelines on HIV self‐testing and partner notification: Supplement to consolidated guidelines on HIV testing services. Geneva: WHO; 2016. 27977094

[11] Thirumurthy H , Masters SH , Mavedzenge SN , Maman S , Omanga E , Agot K . Promoting male partner HIV testing and safer sexual decision making through secondary distribution of self‐tests by HIV‐negative female sex workers and women receiving antenatal and post‐partum care in Kenya: a cohort study. Lancet HIV. 2016;3(6):e266–74. 27240789 10.1016/S2352-3018(16)00041-2 PMC5488644

[12] National AIDS and STI . Control programme (NASCOP). HIV self‐testing: An Operational manual for the delivery of HIV self‐testing services in Kenya. Nairobi, Kenya: NASCOP; 2017.

[13] Masters SH , Agot K , Obonyo B , Napierala Mavedzenge S , Maman S , Thirumurthy H . Promoting partner testing and couples testing through secondary distribution of HIV self‐tests: a randomized clinical trial. PLoS Medicine. 2016;13:e1002166. 27824882 10.1371/journal.pmed.1002166 PMC5100966

[14] Pintye J , Drake AL , Begnel E , Kinuthia J , Abuna F , Lagat H , et al. Acceptability and outcomes of distributing HIV self‐tests for male partner testing in Kenyan maternal and child health and family planning clinics. AIDS. 2019;33(8):1369–78. 30932954 10.1097/QAD.0000000000002211 PMC6546533

[15] Napierala S , Desmond N , Kumwenda M , Tumushime M , Sibanda EL , Indravudh P , et al. Lessons learned from implementation and scale‐up of HIV self‐testing services for female sex workers, Zimbabwe and Malawi. Bull World Health Organ. 2019;97:764–76. 31673192 10.2471/BLT.18.223560 PMC6802700

[16] Siaya CGo . County integrated development plan 2013–2017. Siaya: County Government of Siaya; 2013.

[17] Siaya CGo . County annual development plan 2016–2017. Siaya: County Government of Siaya; 2015.

[18] NACC, NASCOP . Kenya HIV estimates report. Nairobi, Kenya: NACC; 2018.

[19] Kiwanuka N , Ssetaala A , Nalutaaya A , Mpendo J , Wambuzi M , Nanvubya A , et al. High incidence of HIV‐1 infection in a general population of fishing communities around lake Victoria, Uganda. PLoS ONE. 2014;9:e94932. 24866840 10.1371/journal.pone.0094932 PMC4035272

[20] Asiki G , Mpendo J , Abaasa A , Agaba C , Nanvubya A , Nielsen L , et al. HIV and syphilis prevalence and associated risk factors among fishing communities of Lake Victoria, Uganda. Sex Transm Infect. 2011;87(6):511–5. 21835763 10.1136/sti.2010.046805

[21] Cassels S , Camlin CS . Geographical mobility and heterogeneity of the HIV epidemic. Lancet HIV. 2016;3(8):e339–41. 27470023 10.1016/S2352-3018(16)30048-0 PMC5749408

[22] Camlin CS , Kwena ZA , Dworkin SL , Cohen CR , Bukusi EA . “She mixes her business”: HIV transmission and acquisition risks among female migrants in western Kenya. Soc Sci Med. 2014;102:146–56. 24565152 10.1016/j.socscimed.2013.11.004 PMC3935174

[23] Kwena Z , Mwanzo I , Shisanya C , Camlin C , Turan J , Achiro L , et al. Predictors of extra‐marital partnerships among women married to fishermen along lake Victoria in Kisumu County, Kenya. PLoS ONE. 2014;9:e95298. 24747951 10.1371/journal.pone.0095298 PMC3991629

[24] Camlin CS , Kwena ZA , Dworkin SL . Jaboya vs. Jakambi: status, negotiation, and HIV risks among female migrants in the "sex for fish" economy in Nyanza Province, Kenya. AIDS Educ Prev. 2013;25(3):216–31. 23631716 10.1521/aeap.2013.25.3.216 PMC3717412

[25] National AIDS and STI Control Programme . Guidelines for HIV testing services in Kenya. Nairobi: National AIDS Control Programme; 2015.

[26] UNAIDS and STRIVE . Transactional sex and HIV risk: from analysis to action. Geneva: Joint United Nations Programme on HIV/AIDS; 2018.

[27] Maman S , Murray KR , Napierala Mavedzenge S , Oluoch L , Sijenje F , Agot K , et al. A qualitative study of secondary distribution of HIV self‐test kits by female sex workers in Kenya. PLoS ONE. 2017;12:e0174629. 28346527 10.1371/journal.pone.0174629 PMC5367822

[28] Gichangi A , Wambua J , Mutwiwa S , Njogu R , Bazant E , Wamicwe J , et al. Impact of HIV self‐test distribution to male partners of ANC clients: results of a randomized controlled trial in Kenya. J Acquir Immune Defic Syndr. 2018;79(4):467–73. 30148731 10.1097/QAI.0000000000001838 PMC6250253

[29] Cambiano V , Johnson CC , Hatzold K , Terris‐Prestholt F , Maheswaran H , Thirumurthy H , et al. The impact and cost‐effectiveness of community‐based HIV self‐testing in sub‐Saharan Africa: a health economic and modelling analysis. J Int AIDS Soc. 2019;Suppl 1:e25243. 30907498 10.1002/jia2.25243 PMC6432108

[30] Cambiano V , Sibanda E , Morrison M , Shewchuck T , Garnett G , Thirumurthy H , et al. Value of secondary distribution of HIV self‐test kits to male partners of female sex workers. 10th IAS Conference on HIV Science, Mexico City, Mexico; 2019.

[31] Cherutich P , Kurth A , Musyoki H , Nduku K , Maina W . HIV self‐testing in sub‐Saharan Africa: strategies to enhance and measure linkage to care. Retrovirology. 2014;6:23–8.

[32] MacPherson P , Lalloo DG , Webb EL , Maheswaran H , Choko AT , Makombe SD , et al. Effect of optional home initiation of HIV care following HIV self‐testing on antiretroviral therapy initiation among adults in Malawi: a randomized clinical trial. JAMA. 2014;312(4):372–9. 25038356 10.1001/jama.2014.6493 PMC4118051

